# Effect of bone cement augmentation with different configurations of the dual locking plate for femoral allograft fixation: finite element analysis and biomechanical study

**DOI:** 10.1186/s13018-023-03894-3

**Published:** 2023-06-03

**Authors:** Taweechok Wisanuyotin, Permsak Paholpak, Winai Sirichativapee, Wilasinee Sirichativapee, Weerachai Kosuwon

**Affiliations:** Department of Orthopaedics, Faculty of Medicine, Khon Kaen University, Khon Kaen, 40002 Thailand

**Keywords:** Bone cement, Allograft, Femur, Fixation, Biomechanical study, Finite element analysis

## Abstract

**Aims:**

Implant failure in allograft reconstruction is one of the most common problems after treating a large bone defect for a primary bone tumor. The study aimed to investigate the effect of bone cement augmentation with different configurations of dual locking plates used for femoral allograft fixation.

**Methods:**

Four finite element (FE) models of the femur with a 1-mm bone gap were developed at the midshaft with different configurations of the 10-hole fixation dual locking plate (LP) with and without intramedullary bone cement augmentation. Model 1 was the dual LP at the lateral and medial aspect of the femur. Model 2 was Model 1 with bone cement augmentation. Model 3 was the dual LP at the anterior and lateral aspect of the femur. Finally, Model 4 was Model 3 with bone cement augmentation. All models were tested for stiffness under axial compression as well as torsional, lateral–medial, and anterior–posterior bending. In addition, the FE analyses were validated using biomechanical testing on a cadaveric femur.

**Results:**

Model 2 had the greatest axial compression stiffness, followed by Models 1, 4, and 3. Bone cement augmentation in Models 2 and 4 had 3.5% and 2.4% greater axial stiffness than the non-augmentation Models 1 and 3, respectively. In the bone cement augmentation models, Model 2 had 11.9% greater axial compression stiffness than Model 4.

**Conclusion:**

The effect of bone cement augmentation increases construct stiffness less than the effect of the dual LP configuration. A dual lateral–medial LP with bone cement augmentation provides the strongest fixation of the femur in terms of axial compression and lateral bending stiffness.

## Introduction

Reconstruction of a bone defect after wide resection of primary malignant bone tumors remains a challenge for the orthopedic oncologist surgeons. Allograft is widely used for this procedure; however, a high complication rate has been reported, including nonunion, fracture of the graft, and failure of the implant [1]. These complications occur due to a non-vascularized bone graft, which needs more time than normal bone for bone union [2]. Rigid fixation with plate and screws was proposed as the fixation of choice to reduce such complications [3]. The most common site for reconstruction with allograft is the femur [4, 5], and the best choice for rigid fixation is a locking plate (LP) [6]. Various methods have been proposed to reduce complications and improve construct strength, including dual LP fixation to increase construct stiffness [7–10] and augmentation of bone cement to improve the mechanical properties of the allografts [1, 11].

Polymethylmethacrylate (PMMA) bone cement has been used in orthopedic surgery since 1945 [12]. Gerrand et al. [1] retrospectively reviewed the addition of intramedullary bone cement to large segment bone allografts and found that graft survival improved with a decreasing risk of fracture. The advantage of reinforcing with bone cement is that it improves the mechanical properties of allografts and leads to a lower fracture rate. Gupta et al. [10] reported that 46 patients underwent reconstruction with an intercalary allograft of the femur, tibia, and humerus. The overall survival was 84.8% and 33% (15 patients), which had a complication. Gupta et al. [10] concluded that intercalary allografts augmented with intramedullary cement with compression plate fixation provide a reliable and durable reconstruction method after the excision of a primary diaphyseal bone tumor. Ozaki et al. [13] retrospectively reviewed the allograft reconstruction after resection of bone sarcomas and compared the outcomes of allograft reconstruction of 26 intramedullary cemented massive allografts with 19 allografts without cementation. The allograft was fractured in 3 cases in the uncemented group, while no graft fracture occurred in the cemented group. Late infections developed in 1 of the cemented group and in 4 of the uncemented group. Ozaki et al. [13] concluded that intramedullary graft cementation trends to reduce fracture and infection rates.

No study to date has identified the role of augmenting bone cement with dual LP for femoral allograft fixation. A previous study showed that a lateral and medial 10-hole LP configuration provided the most rigid and strongest fixation [7]. However, the main problem encountered while performing this procedure was screw trajectory of the opposing screws in the medial–lateral direction. We hypothesized that bone cement augmentation of the dual LP in the orthogonal direction (lateral–anterior)—technically easier to perform than the former dual LP configuration—should result in greater than or equal to construct strength than dual LP in the lateral and medial configuration.

We thus conducted the current study to investigate the biomechanical properties of the dual LP for femoral allograft between the orthogonal (anterior and lateral) and opposing direction (medial and lateral) both with and without bone cement augmentation.

## Material and methods

The Institutional Review Board of Khon Kaen University, Khon Kaen, Thailand, approved the study (HE641110). We first performed the finite element (FE) analysis of the femur, followed by the biomechanical study of the cadaveric femurs. For the results of an FE analysis to be credible, the model must be experimentally validated in at least one load case [14].

### FE analysis

The DICOM format of the computed tomography (CT) scan of the fresh femoral cadaver was used to create the femoral models, which were then exported to MIMICS 10.01 (The Materialise, Leuven, Belgium). These models were then imported into SolidWorks 2015 software (SolidWorks Corp., MA, USA) and PowerShape 2013 (Autodesk Inc., San Rafael, California, USA). Stiffness analysis of the plate assembly was performed using ANSYS workbench 15.1 (ANSYS, Inc., Canonsburg, Pennsylvania, USA).

All the FE models were meshed using solid tetrahedral elements with ten nodes. The mesh of the bone model was refined with an element size of 4.0 mm. The model comprised the number of nodes and elements (924,099 and 593,835, respectively). The bones, implants, and bone cement material properties were assumed to be isotropic and linearly elastic. The Poisson’s ratio of the femur was 0.3 with a Young’s modulus of 0.805 GPa. The femur was 27.7 mm in diameter and 430 mm in length. The length of the 10-hole locking plates was 186 mm. The respective width and thickness of the locking plate were 17.5 and 6 mm. The screw was 4.5 mm in diameter and 33 mm in length. The locking plate and screws were stainless steel, element size 1 mm with a Young’s modulus of 200 GPa. The polymethylmethacrylate (PMMA) bone cement had a Young’s modulus of 2.7 GPa and a Poisson’s ratio of 0.35 [15]. The interface condition between the bone and plate was set as the contact condition, while the interface between the screw and the plate and the screw and the bone represented the bonded condition. The friction coefficient between the bone–cement interface was 0.3 [16].

**Fig. 1:**
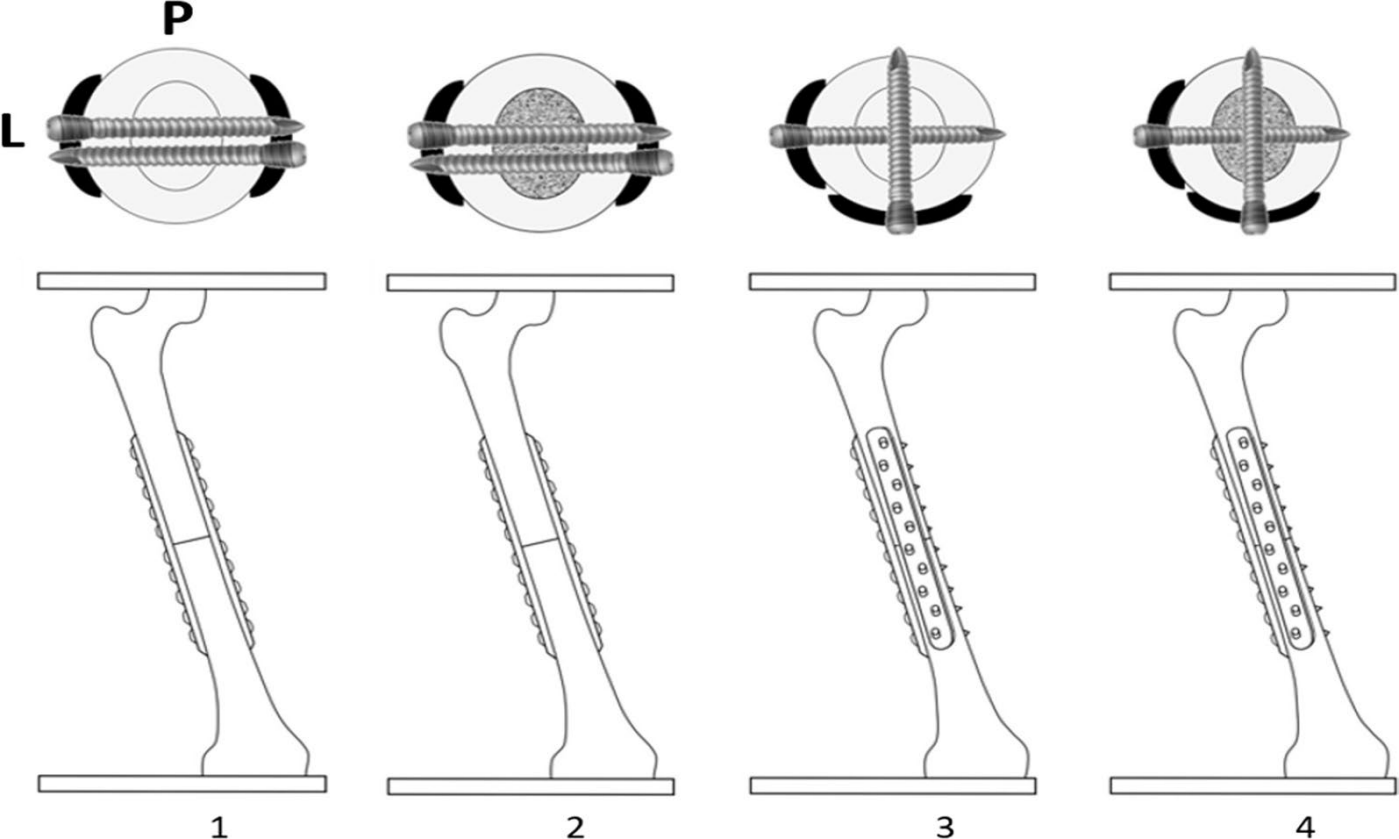
FE models of femurs with 10-hole dual LP configurations (P = posterior, L = lateral)

Four FE models with a bone gap of 1 mm were created at the midshaft of the femur to simulate the host–graft junction [17, 18] (Fig. 1). The proximal part of the femur represented as the host bone, while the distal femur represented as the femoral allograft. In Model 1, the 10-hole locking plates were placed at the lateral and medial aspect of the femur. Model 2 was Model 1 with bone cement augmentation. Model 3 was the dual LP at the anterior and lateral aspect of the femur. Finally, Model 4 was Model 3 with bone cement augmentation.

**Fig. 2:**
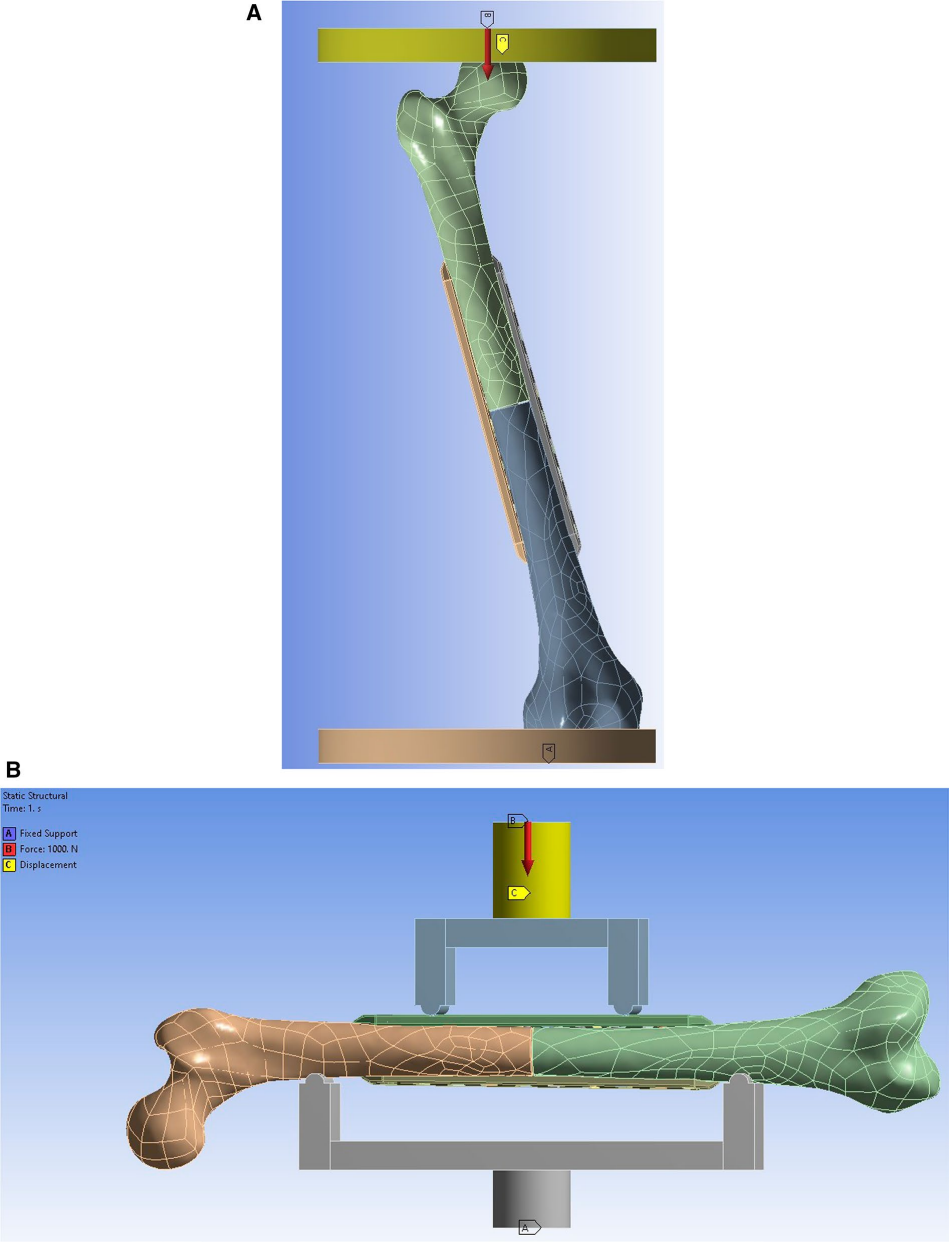
FE model of the 10-hole dual LP during (**A**) the axial compression and torsional test and (**B**) the LM bending test

The femurs were tested for axial compression and torsional stiffness in the one-legged stance phase of walking with 15 degrees of adduction in the coronal plane and aligned vertically in the sagittal plane [19, 20] (Fig. 2A). A force of 1500 N was applied to the center of the femoral head to test for axial compression stiffness.

As for four-point bending stiffness, the femurs were positioned as shown in Fig. 2B, and a 1000-N force was applied in the lateral–medial (LM) and anterior–posterior (AP) direction to assess LM and AP bending stiffness, respectively.

As for torsional stiffness testing, the machine was set to torque at 12 N·m with a frequency of 0.1 Hz in three cycles. Then, the force was applied to the femoral head using angular displacement control (0.1 degrees/s) for both external and internal rotations.

The stiffness of each model was calculated by dividing the maximum force applied by the maximum deformation. The deformation (strain) measurements were recorded: between 10 and 1500 N as applied axially for axial compression testing; between 0.1 and 12 N·m for torsional testing; and between 10 and 1000 N for LM and AP bending.

### Biomechanics testing

The fresh-frozen human cadaveric femur was obtained from the Department of Anatomy, Khon Kaen University. A 75-year-old man donor consented to using his body for research purposes. The femur was radiographed before the study to exclude fractures, infections, and bone tumors. All soft tissues were stripped from the bone. The femur was thawed at room temperature for 12 h before applying the plate and screws. An intramedullary canal was reamed 2 mm larger than the diameter of each femur. A reciprocating saw was used to make a 1-mm transverse osteotomy at midshaft of the femur to mimic the host–graft junction. Plastic cement plugs were inserted 95 mm from the osteotomy site at both the proximal and distal parts of the femur.

**Fig. 3:**
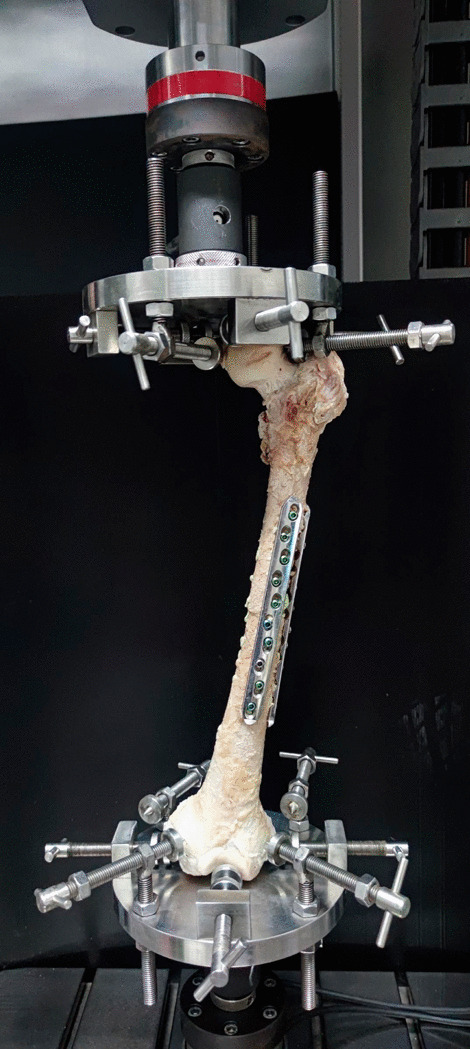
Biomechanical testing for axial compression and torsional testing with the femur at 15 degrees of adduction in the coronal plane aligned vertically in the sagittal plane. The femoral head and condyle were secured to the Instron ElectroPuls ™ E10000 Linear-Torsion with custom, multiple, adjustable jigs

Polymethylmethacrylate (PMMA) bone cement (Rally®, medium viscosity with Gentamicin sulfate, Smith & Nephew, Inc., Memphis, TN, USA) was hand-mixed and left to cure at room temperature. The cement was poured into the medullary region of the femur, at both the proximal and distal segments. The cement was allowed to set for 20 min. The locking plates (4.5/5.0, broad Stainless-Steel Locking Compression Plate (LCP) System, DePuy Synthes Raynham, MA, USA) were applied laterally and anteriorly to the femurs according to standard surgical procedure. The femoral head and condyle were secured with multiple, custom, adjustable jigs to an Instron ElectroPuls™ E10000 Linear-Torsion (Illinois Tool Works Inc, MA, USA) (Fig. 3). Before biomechanical testing, the femurs were stored at room temperature for 6 h to allow cement curing. The axial compression, four-point bending, and torsional testing were performed three times to ensure reproducibility.

### Axial compression testing

In order to simulate the single-leg stance phase of walking, an axial compression load of 1500 N was used with a preload of 50 N [19]. Finally, a maximum load of 1500 N was applied at 100 N/s. Bluehill 3 software was used to run all the tests.

### Four-point bending test

The four-point bending test was done in two directions, LM and AP, to assess the respective LM and AP bending stiffness. The center of the machine was placed at the fracture site. A preload of 50 N was applied to a maximum of 1000 N at 100 N/s.

### Torsional testing

The femur was positioned similarly for axial compression testing. A torque of 12 N·m at a frequency of 0.1 Hz was tested in both the internal and external rotations.

### Statistical analyses

The Pearson’s correlation coefficient was used to compare the FE analysis results and the biomechanical testing. A *p*-value of less than 0.05 was considered statistically significant (SPSS version 23, SPSS Inc., Chicago, IL, USA).

## Results

### FE analysis

**Fig. 4:**
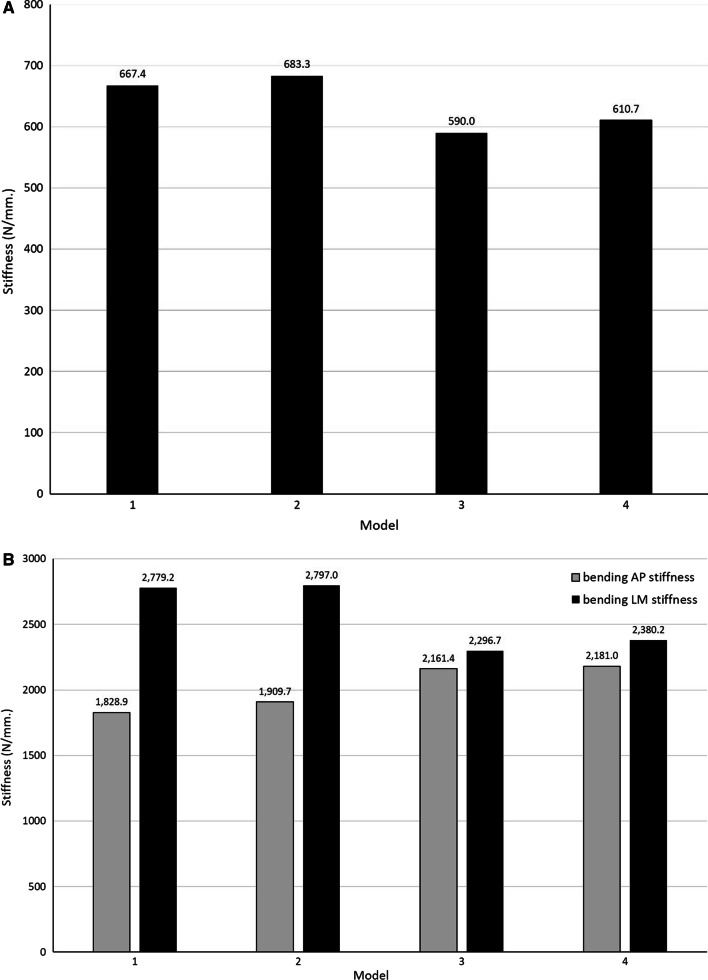

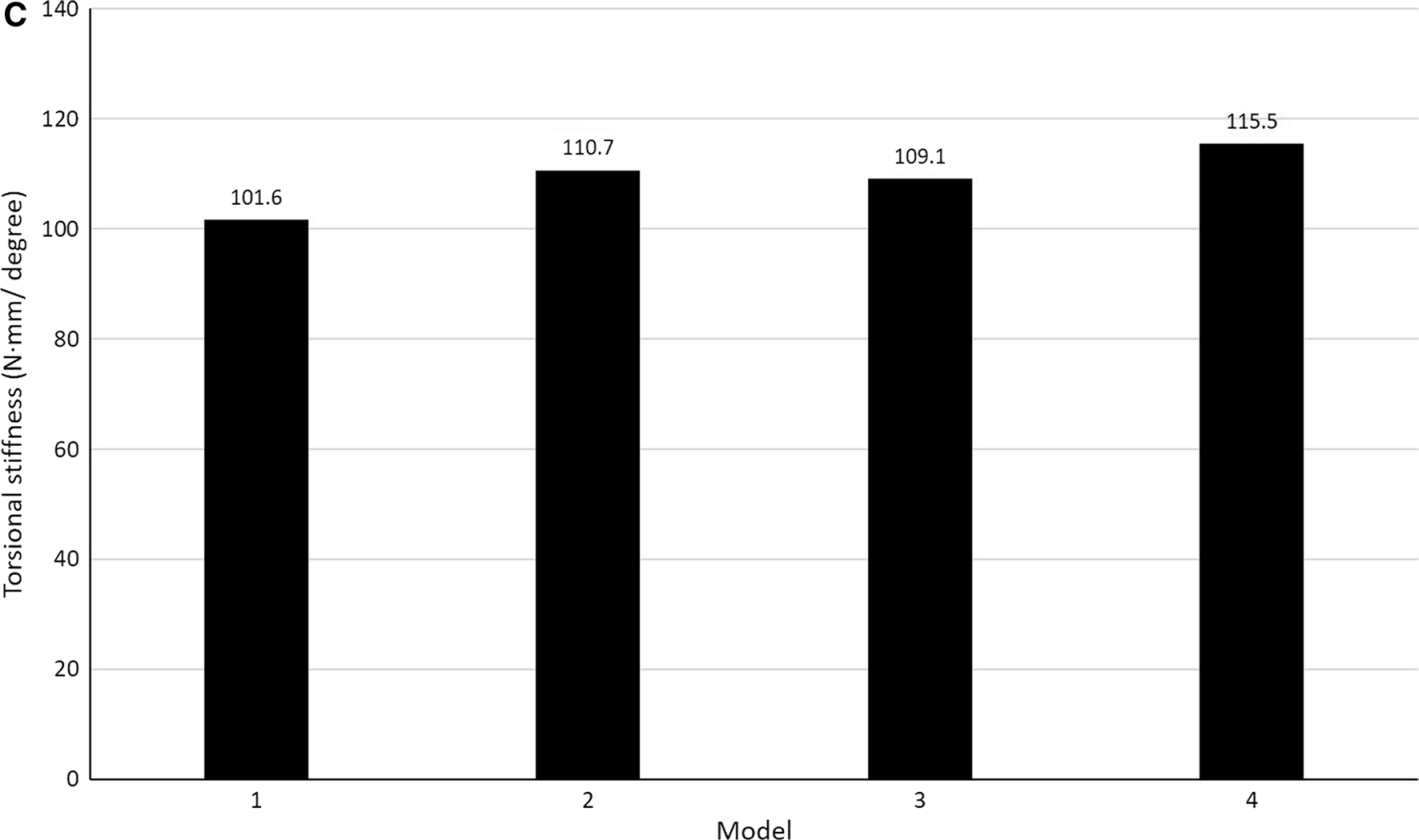
FE analysis of dual LP (**A**) axial compression stiffness (**B**) bending stiffness (**C**) torsional stiffness

The axial compression, AP and LM bending, and torsional stiffness for Models 1, 2, 3, and 4 are presented in Fig. 4A–C, respectively.

Model 2 provided the greatest axial compression and LM bending stiffness, while Model 4 provided the greatest AP bending and torsional stiffness.

As for the effect of bone cement for the same configuration of dual LP (Model 1 vs. Model 2 and Model 3 vs. Model 4), Model 2 over against Model 1 had 2.4%, 4.4%, 0.6%, and 8.9% greater axial compression, AP bending, LM bending, and torsional stiffness, respectively. Meanwhile, Model 4 over against Model 3 had 3.5%, 0.9%, 3.6%, 5.8% greater axial compression, AP bending, LM bending, and torsional stiffness, respectively.

As for the effect of a different dual LP configuration for the model with bone cement (Model 2 vs. Model 4), Model 2 had 11.9% and 17.5% greater axial compression stiffness and LM bending than Model 4, respectively. On the other hand, Model 4 had greater AP bending and torsional stiffness than Model 2 for 14.2% and 4.3%, respectively.

### Biomechanical testing

The axial compression, AP, LM bending and torsional stiffness of the fresh-frozen femurs of Model 4 are 614.1 ± 5.2 N/mm, 2215.9 ± 2.6 N/mm, 2734.3 ± 11.4 N/mm and 126.8 ± 1.9 N·m/degree, respectively.

### Validation of FE analysis and biomechanical testing

**Fig. 5:**
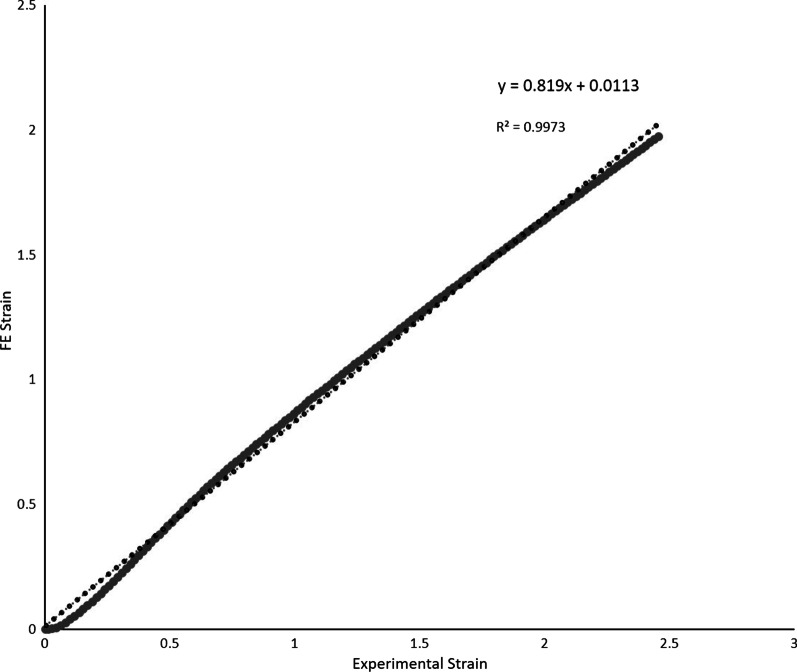
Validation of the FE strains and experimental strains

A strong correlation between the FE analysis and the biomechanical testing was demonstrated (Fig. 5). The Pearson correlation was *r*^2^ = 0.99, *p* < 0.01.

## Discussion

Our previous study on the configuration of the dual LP concluded that the dual LP in the medial and lateral aspect of the femur provided the highest axial compression stiffness. The construction with high stiffness means that it has immediate stability after surgery and can be considered optimal [19, 21].

The FE analysis is a computational tool for comparative studies of various implant designs [21, 22]. Cadaveric studies reveal that specimens vary in geometry and mechanical properties, which is difficult to reproduce [23]. In the current study, the FE analysis was used to test the different configurations of dual LP for femoral fixation with and without bone cement augmentation. The results of a FE analysis are validated by biomechanics testing to ensure their credibility. [14, 19, 21, 24, 25].

The effect of plate configuration for femoral shaft fixation has been investigated. El Beaino et al. [9] compared the mechanical properties of conventional and locking dual plates in adjacent and orthogonal orientations for fixation of femoral shaft fractures. El Beaino et al. [9] found that dual LP provides higher stability than their dual conventional plates and that orthogonal dual plate configuration is more stable and biomechanically superior to dual adjacent plating. Our previous study reported that the dual LP configuration with 10-hole medial and lateral LP provided the femur's most rigid and strongest fixation [7].

Mechanical properties of bone cement were investigated, and weak in tensile strength but strong in compressive strength were observed [15, 26]. Sas et al. [27] studied the effect of cement augmentation of metastatic lesions in the proximal femur and reported that cement augmentation did not significantly increase stiffness. Wahnert et al. [28] studied the implant augmentation with bone cement in osteoporotic distal femur fractures and found that there was no significant difference in axial compression stiffness between the cement augmentation and non-augmentation groups. These results agree with our findings that bone cement results in a 2.4% increase in axial compression stiffness in models with a dual medial–lateral locking plate versus 3.5% in models with a dual anterior–lateral locking plate.

The dual LP configuration had a greater impact on axial compression stiffness than bone cement augmentation with respect to construct stiffness. The current study revealed that bone cement augmentation models provided a 0.6–8.9% increment in construction stiffness than non-cement augmentation models. In bone cement augmentation models, differences in the dual LP configuration resulted in a 4.3–17.5% increase in construction stiffness, which could be explained by the Young’s modulus of locking plate being greater than that of bone cement.

Model 4 was the most favorable technique and relatively easy to perform: it was achieved with a dual LP configuration in the orthogonal direction (anterior and lateral) augmented with bone cement; in contrast to a dual LP in the opposing direction (medial and lateral) with a screw trajectory toward the opposite side in the medial–lateral direction. Based on our results, Model 4 had greater AP bending and torsional stiffness than Model 2, while Model 2 had greater axial compression stiffness and LM bending stiffness than Model 4. We suggest the optimum dual LP configuration is Model 2 because the failure of the locking plate for femoral fixation usually fails due to an axial load [29, 30].

The limitations of this study are: (1) the study focused on short-term results of bone cement, so long-term outcomes need to be investigated in a prospective clinical study; (2) the study accounted only for bone and no soft tissue was considered, so the effect of bone cement augmentation in patients may be different; and (3) the effect of temperature of the bone cement was not included, which could affect the results.

## Conclusion

For fixation of femoral allograft, dual LP in the LM configuration with bone cement augmentation provided the strongest fixation of the femur. However, the plate configuration has a greater effect than the augmentation of bone cement on axial compression stiffness. We thus recommend using bone cement augmentation with dual LP in the LM configuration for femoral allograft reconstruction.

## Data Availability

The datasets used and/or analyzed during the current study are available from the corresponding author on reasonable request.

## References

[1] Gerrand CH, Griffin AM, Davis AM, Gross AE, Bell RS, Wunder JS (2003). Large segment allograft survival is improved with intramedullary cement. J Surg Oncol.

[2] Frisoni T, Cevolani L, Giorgini A, Dozza B, Donati DM (2012). Factors affecting outcome of massive intercalary bone allografts in the treatment of tumours of the femur. J Bone Jt Surg Br Vol.

[3] Bus MP, Dijkstra PD, van de Sande MA, Taminiau AH, Schreuder HW, Jutte PC (2014). Intercalary allograft reconstructions following resection of primary bone tumors: a nationwide multicenter study. J Bone Jt Surg Am.

[4] Sorger JI, Hornicek FJ, Zavatta M, Menzner JP, Gebhardt MC, Tomford WW (2001). Allograft fractures revisited. Clin Orthop Relat Res.

[5] Bus MP, van de Sande MA, Taminiau AH, Dijkstra PD (2017). Is there still a role for osteoarticular allograft reconstruction in musculoskeletal tumour surgery? A long-term follow-up study of 38 patients and systematic review of the literature. Bone Jt J.

[6] Strauss EJ, Schwarzkopf R, Kummer F, Egol KA (2008). The current status of locked plating: the good, the bad, and the ugly. J Orthop Trauma.

[7] Wisanuyotin T, Sirichativapee W, Paholpak P, Kosuwon W, Kasai Y (2020). Optimal configuration of a dual locking plate for femoral allograft or recycled autograft bone fixation: a finite element and biomechanical analysis. Clin Biomech.

[8] Buecker PJ, Berenstein M, Gebhardt MC, Hornicek FJ, Mankin HJ (2006). Locking versus standard plates for allograft fixation after tumor resection in children and adolescents. J Pediatr Orthop.

[9] El Beaino M, Morris RP, Lindsey RW, Gugala Z (2019). Biomechanical evaluation of dual plate configurations for femoral shaft fracture fixation. Biomed Res Int.

[10] Gupta S, Kafchinski LA, Gundle KR, Saidi K, Griffin AM, Wunder JS (2017). Intercalary allograft augmented with intramedullary cement and plate fixation is a reliable solution after resection of a diaphyseal tumour. Bone Jt J.

[11] Donati D, Capanna R, Campanacci D, Del Ben M, Ercolani C, Masetti C (1993). The use of massive bone allografts for intercalary reconstruction and arthrodeses after tumor resection: A multicentric European study. La Chirurgia degli organi di movimento.

[12] Webb JC, Spencer RF (2007). The role of polymethylmethacrylate bone cement in modern orthopaedic surgery. J Bone jt Surg Br Vol.

[13] Ozaki T, Hillmann A, Bettin D, Wuisman P, Winkelmann W (1997). Intramedullary, antibiotic-loaded cemented, massive allografts for skeletal reconstruction: 26 cases compared with 19 uncemented allografts. Acta Orthop Scand.

[14] Cheung G, Zalzal P, Bhandari M, Spelt JK, Papini M (2004). Finite element analysis of a femoral retrograde intramedullary nail subject to gait loading. Med Eng Phys.

[15] Khellafi H, Bouziane MM, Djebli A, Mankour A, Bendouba M, Bouiadjra BB (2019). Investigation of mechanical behaviour of the bone cement (PMMA) under combined shear and compression loading. J Biomim Biomater Bi.

[16] Janssen D, Mann KA, Verdonschot N (2009). Finite element simulation of cement-bone interface micromechanics: a comparison to experimental results. J Orthop Res.

[17] Egol KA, Kubiak EN, Fulkerson E, Kummer FJ, Koval KJ (2004). Biomechanics of locked plates and screws. J Orthop Trauma.

[18] Ganesh VK, Ramakrishna K, Ghista DN (2005). Biomechanics of bone-fracture fixation by stiffness-graded plates in comparison with stainless-steel plates. Biomed Eng Online.

[19] Ebrahimi H, Rabinovich M, Vuleta V, Zalcman D, Shah S, Dubov A (2012). Biomechanical properties of an intact, injured, repaired, and healed femur: an experimental and computational study. J Mech Behav Biomed Mater.

[20] Dubov A, Kim SY, Shah S, Schemitsch EH, Zdero R, Bougherara H (2011). The biomechanics of plate repair of periprosthetic femur fractures near the tip of a total hip implant: the effect of cable-screw position. Proc Inst Mech Eng H.

[21] Coquim J, Clemenzi J, Salahi M, Sherif A, Tavakkoli Avval P, Shah S (2018). Biomechanical analysis using FEA and experiments of metal plate and bone strut repair of a femur midshaft segmental defect. Biomed Res Int.

[22] Chen SH, Chiang MC, Hung CH, Lin SC, Chang HW (2014). Finite element comparison of retrograde intramedullary nailing and locking plate fixation with/without an intramedullary allograft for distal femur fracture following total knee arthroplasty. Knee.

[23] Papini M, Zdero R, Schemitsch EH, Zalzal P (2007). The biomechanics of human femurs in axial and torsional loading: comparison of finite element analysis, human cadaveric femurs, and synthetic femurs. J Biomech Eng.

[24] Wako Y, Nakamura J, Matsuura Y, Suzuki T, Hagiwara S, Miura M (2018). Finite element analysis of the femoral diaphysis of fresh-frozen cadavers with computed tomography and mechanical testing. J Orthop Surg Res.

[25] Trabelsi N, Yosibash Z, Wutte C, Augat P, Eberle S (2011). Patient-specific finite element analysis of the human femur–a double-blinded biomechanical validation. J Biomech.

[26] Saha S, Pal S (1984). Mechanical properties of bone cement: a review. J Biomed Mater Res.

[27] Sas A, Van Camp D, Lauwers B, Sermon A, van Lenthe GH (2020). Cement augmentation of metastatic lesions in the proximal femur can improve bone strength. J Mech Behav Biomed Mater.

[28] Wahnert D, Hofmann-Fliri L, Richards RG, Gueorguiev B, Raschke MJ, Windolf M (2014). Implant augmentation: adding bone cement to improve the treatment of osteoporotic distal femur fractures: a biomechanical study using human cadaver bones. Medicine.

[29] Gervais BVA, Raison M, Brochu M (2016). Failure analysis of a 316L stainless steel femoral orthopedic implant. Case Stud Eng Fail Anal.

[30] Duda GN, Schneider E, Chao EY (1997). Internal forces and moments in the femur during walking. J Biomech.

